# The optimal angle of screw for using cement-screw technique to repair tibial defect in total knee arthroplasty: a finite element analysis

**DOI:** 10.1186/s13018-022-03251-w

**Published:** 2022-07-26

**Authors:** Guanghui Zhao, Shuxin Yao, Jianbing Ma, Jianpeng Wang

**Affiliations:** Department of Joint Surgery, Honghui Hospital, Xi’An Jiaotong University, No. 555 East Youyi Road, Xi’an, Shanxi China

**Keywords:** Cement-screw technique, Bone defect, Total knee arthroplasty, Finite element analysis

## Abstract

**Background:**

The cement-screw technique is a convenient method to repair tibial plateau defects in primary and revision total knee arthroplasty (TKA). However, the optimal angle of screw insertions is unknown. This study aimed to perform a finite element analysis (FEA) to determine the optimal screw angle for the repair of tibial plateau defects in TKA.

**Methods:**

Seven FEA models were set and two common different defects (defect 1: area < 12%, depth < 12 mm; defect 2: area > 12%, depth > 12 mm) were simulated. One screw was used in defect 1, and one or two screws were used in defect 2. Screws were parallel to the proximal cortical bone (oblique screw) or perpendicular to the upper surface (vertical screw) of the tibia. Contact stresses on cancellous bone in different areas were determined. Maximum principal stress on the cancellous bone around each screw was also compared.

**Results:**

The FEA models showed that stresses on the surface of cancellous bone in tibial defect (0.13–0.39 MPa) and stress focus spot (0.45 MPa) around the screw were lower when one vertical screw was used in defect 1. The stresses on the surface of cancellous bone in tibial defect (0.09–0.44 MPa), stresses in the medial tibial plateau (0.14–0.21 MPa), and stress focus spot around the screws were lowest (0.42 MPa and 1.37 MPa) when two vertical screws were used in defect 2, followed by of one vertical and one oblique (0.16–0.48 MPa; 0.15–0.21 MPa; 1.63 MPa and 1.11 MPa). No other statistically significant differences were found.

**Conclusions:**

Either for one or two screws, those perpendicular to the upper surface achieve better stability than those parallel to the proximal cortical bone of the tibia. If two vertical screws cannot be performed, one vertical and one oblique is also acceptable.

## Background

The cement-screw technique has been used to repair tibial bone defects in total knee arthroplasty (TKA) for over 40 years [1]. The cancellous screw is the metal reinforcement used on a defect filled with bone cement. Compared to other techniques, the cement-screw technique is less time-consuming, easier to perform, and less expensive [2, 3]. Although successful short-term and long-term follow-up is reported for the cement-screw technique, it may not be that simple [4–6]. There remains no consensus about the optimal angle of the screws, which is currently based on personal experience [7, 8]. Some have suggested that the screw should be parallel to the proximal cortical bone, while others believe that it should be perpendicular to the upper surface of the tibia plateau. In a finite element analysis (FEA), Zheng et al. [9] assessed the differences between vertical and oblique screws and demonstrated that vertical screws achieve better stability than oblique screws. However, the aim of the study was to determine whether differences exist between vertical and oblique screws and, therefore, only compared the difference for bones with a 12% defect area and a 12-mm depth. Defects with a larger area and depth needing two or more screws, which occurs in clinical practice, were not compared. The authors also did not indicate whether the oblique screw was parallel to the proximal cortical bone of the tibia, similar to previous reports. This study aimed to perform an FEA to determine the optimal screw angle either when one or two screws are used to provide a more basic reference for clinical practice.

## Methods

### Solid model

Using computed tomography (CT) images of a healthy volunteer (female, 20 years old, 165 cm in height, and 50 kg in weight), a three-dimensional (3D) model of the tibia was constructed using Mimics 21.0 (Materialise Company, Leuven, Belgium). The model was imported into Geomagic Studio 12.0 (3D Systems, Inc., North Carolina, USA) for optimization and then into SolidWorks 2016 (Dassault Systems SolidWorks Corp., Waltham, Massachusetts, USA) for creating the bone defect and implanting the prosthesis. Based on previous studies, two types of bone defects (defect 1, 9% (< 12%) of a total plateau in area, 8 mm (< 12 mm) in depth, and defect 2, 18% (> 12%) of a total plateau in area, 15 mm (> 12 mm) in depth) were made after performing a horizontal resection 9 mm above the tibial plateau [9, 10].

### Tibial component

The tibial component consisted of a baseplate and a polyethylene insert. The tibial baseplate was made of cobalt-chrome alloy (#2 size, U2TM Knee, United Orthopedic, Taiwan), and its geometry was obtained by direct measurement. The posterior-stabilized fixed-bearing ultra-high molecular weight polyethylene (UHMWPE) insert was 9 mm in thickness with a rounded-top with flat-inferior surfaces. The tibial component was positioned at the plateau perpendicular to the mechanical axis of tibia.

### Screw and bone cement models

**Fig. 1:**
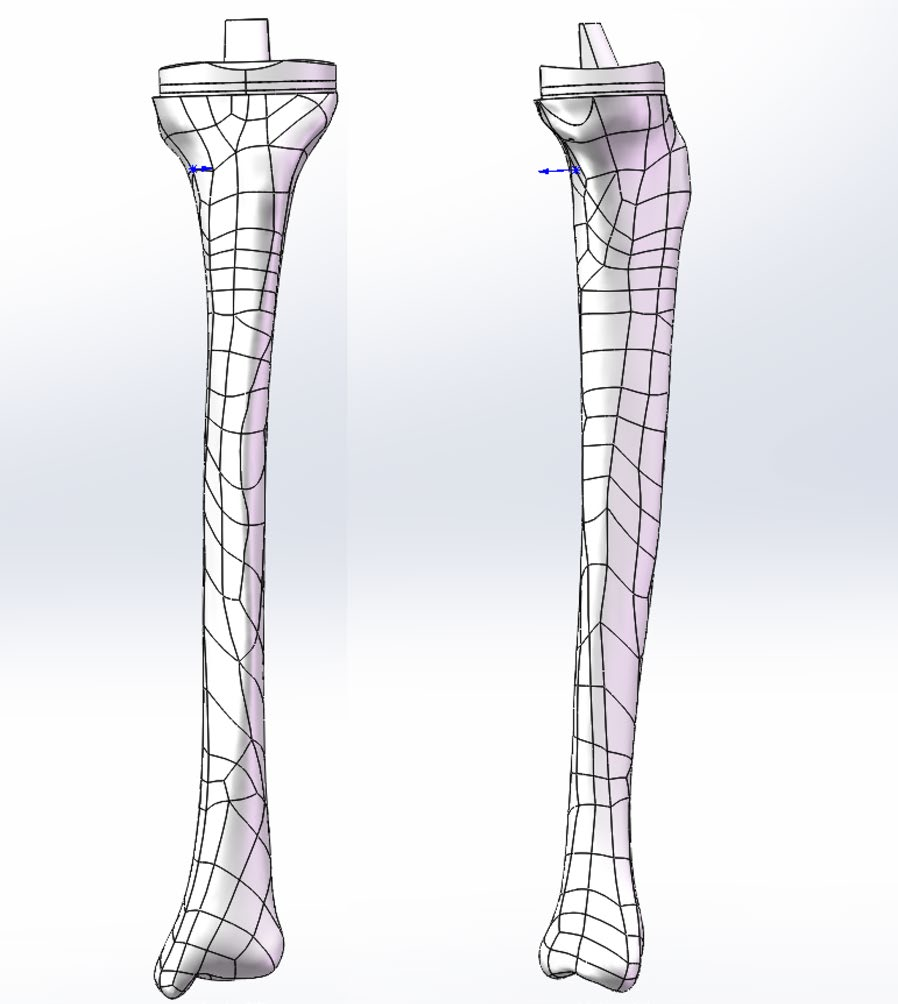
The anteroposterior and lateral view of whole model, consisted of ultrahigh molecular weight polyethylene (UHMWPE) insert, tibial prothesis, cement, screw and tibia

**Fig. 2:**
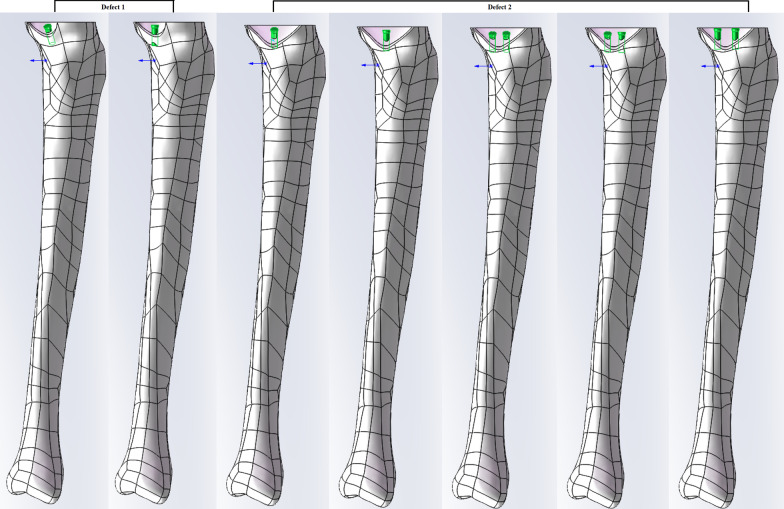
Different tibial bone defects and one or two screws inserted with different angles

In defect 1, a 4 mm × 14 mm cancellous screw (Smith and Nephew, Memphis, TN, USA) was inserted. In defect 2, one or two screws were implanted. A 1.5 mm thick cement layer was used to fix the tibial tray to the upper surface of the tibia after resection [12]. Bone separated from the defect area was defined as the cement model filling it. Finally, all parts were assembled in SolidWorks software (Figs. 1 and 2).

### Material properties and boundary and loading conditions

**Fig. 3:**
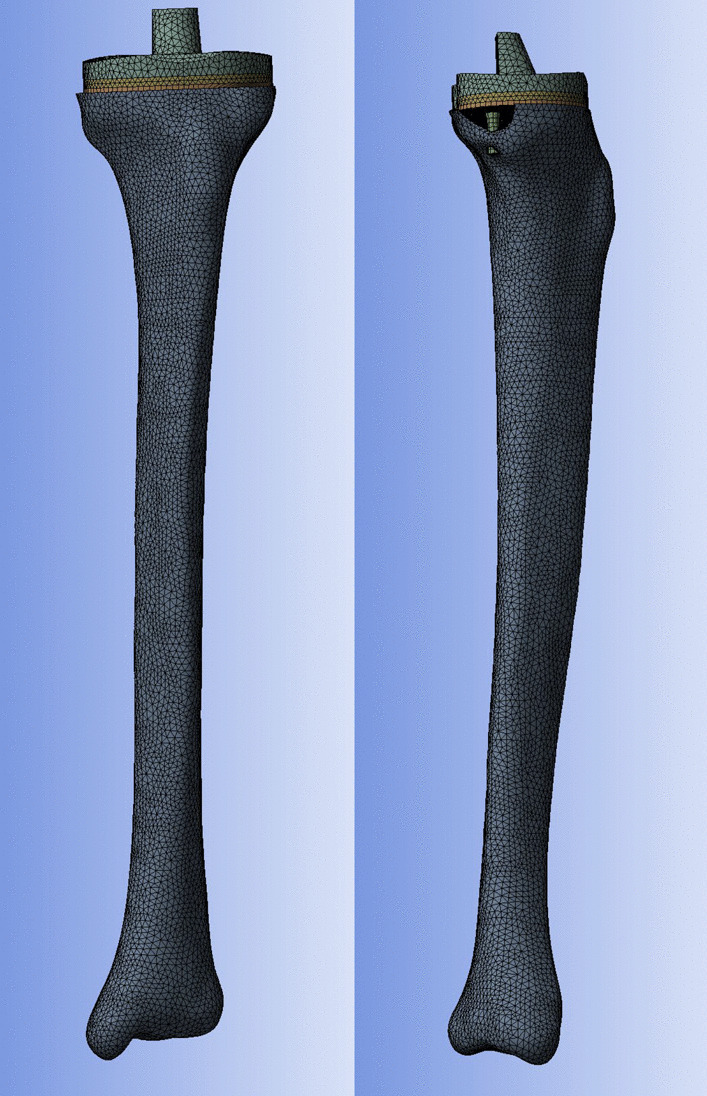
The anteroposterior and lateral view of finite element models of the whole model

**Fig. 4:**
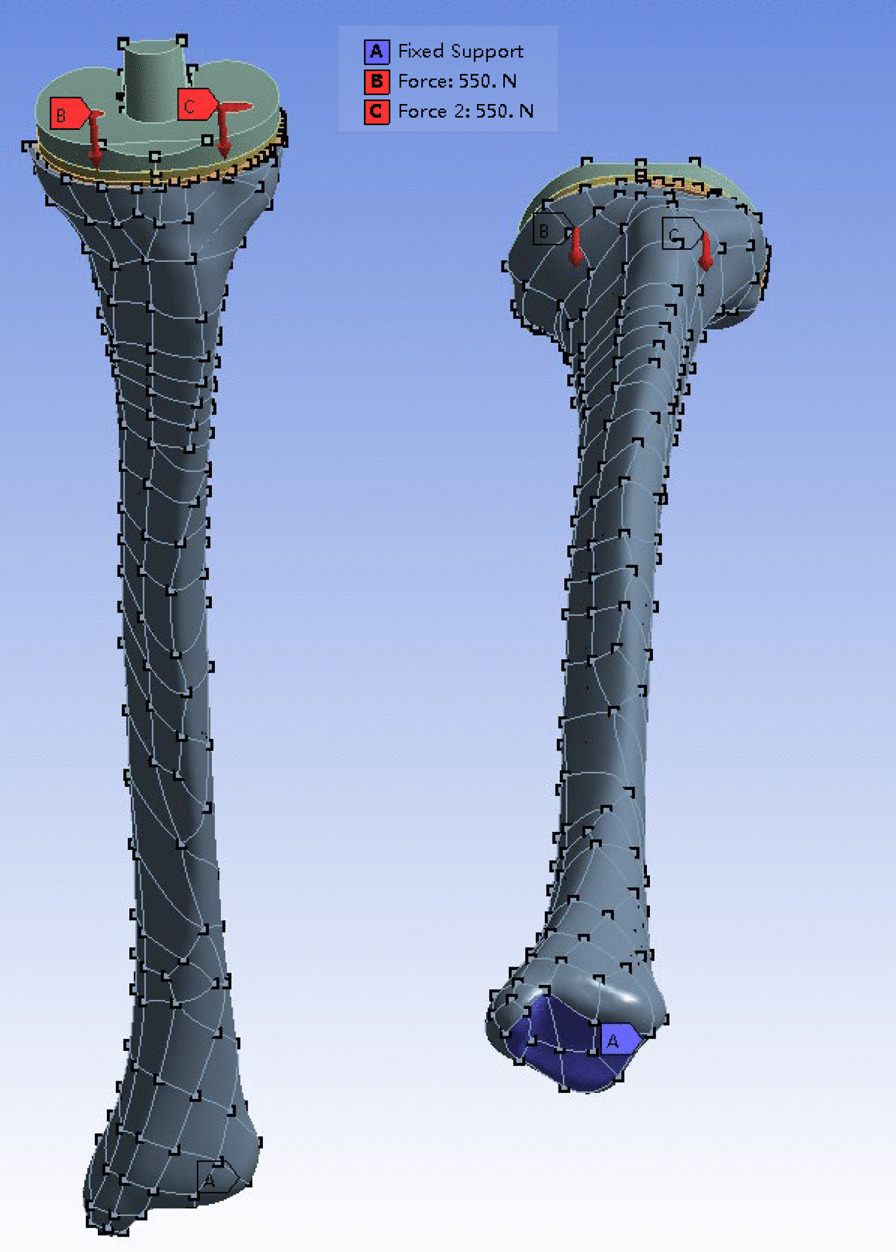
Load areas on the tibial plateau and fixed areas on the distal tibia

For proper meshing and simulation, the models were imported into ANSYS Workbench 17 (Swanson Analysis Systems, Inc., Houston, Pennsylvania, USA) (Fig. 3). The mechanical properties of the component materials are shown in Table 1 [9, 13]. In an uncemented implant, friction is the only mechanism for the transfer of shear stresses at an interface until bone ingrowth occurs; however, friction is expected to play an important role in a cemented implant only after the initiation of interface debonding. Hence, the contact behavior between the screws and bone was defined as the frictional surface-to-surface with a coefficient of 0.3; the others were defined as fully bonded [14, 15]. All implant components were modeled as linear elastic isotropic materials [16]. A total load of 1100N (2.2 times body weight) was used and applied in a 1:1 ratio between the medial and lateral plateaus [17]. The inferior surface of the distal tibia was fixed in all directions (Fig. 4). A conservatively low value for damage stress (2.8 MPa) of the cancellous bone was adopted, and a resorption threshold of 0.1 MPa was used to assess possible bone resorption [18].

**Table 1:** The mechanical properties of component materials in each model

Material	*E* (MPa)	*v*
Cortical bone	17,000	0.3
Cancellous bone	700	0.3
UHMWPE (tibial insert)	2300	0.25
Cobalt-chrome alloy (tibial prothesis)	248,000	0.3
PMMA (cement)	2270	0.46
Titanium alloy (screw)	110,000	0.3

**Fig. 5:**
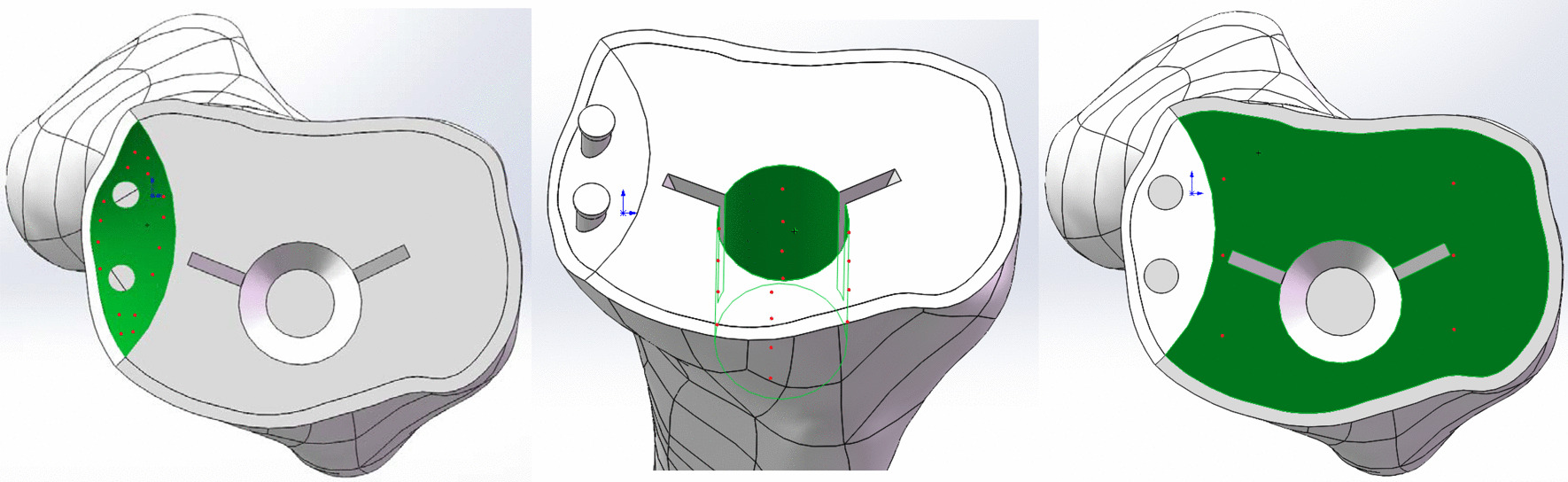
Stresses measured at different points on the surface of defect, one the midcourt line of the medial and lateral plateau, and on the surface of cancellous bone in the medullary cavity

Seven FEA models were set up to determine the differences between screw angles with one or two screws (Table 2). Screws perpendicular to the upper surface of the tibial plateau were defined as vertical; screws parallel to the proximal cortical bone were defined as oblique. The different stresses were compared by measuring 6 points on the midcourt line of the medial and lateral plateaus, 16 points on the surface of cancellous bone in the defect, and 16 points on the surface of cancellous bone in the medullary cavity (Fig. 5). The maximum principal stresses of cancellous bone around each screw were also measured. All data were analyzed using SPSS version 22 (SPSS Inc., Chicago, IL, USA). A Paired Student’s t-test was used to compare the differences in stresses between models, and statistical significance was set at *p* < 0.05.

**Table 2:** The composition of each model

	Model 1	Model 2	Model 3	Model 4	Model 5	Model 6	Model 7
Number of screws	1	1	1	1	2	2	2
Defect area%	9%	9%	18%	18%	18%	18%	18%
Depth (mm)	8	8	15	15	15	15	15

## Results

### Bone defect

**Fig. 6:**
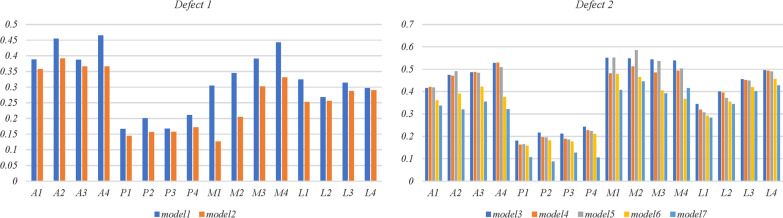
Stresses (MPa) at 16 points on the surface of defects. A: anterior; P: posterior; M: medial; L: lateral

As shown in Fig. 6, defects were divided into 4 sections (anterior, posterior, medial, and lateral), and stresses were measured at 4 points on the surfaces of each. The results showed that stresses were significantly lower in model 2 (0.13–0.39 MPa) than in model 1 (0.17–0.46 MPa) (p < 0.05). When compared to model 3 (0.18–-0.55 MPa), model 4 (0.16–0.53 MPa), or model 5 (0.16–0.58 MPa), stresses were significantly lower in model 6 (0.16–0.48 MPa, *p* < 0.05) and model 7 (0.090.44 MPa, *p* < 0.05). Although stress shielding was possible, stresses measured on the surface of the defect in model 7 were lowest compared to other models.

### Tibial surface

**Fig. 7:**
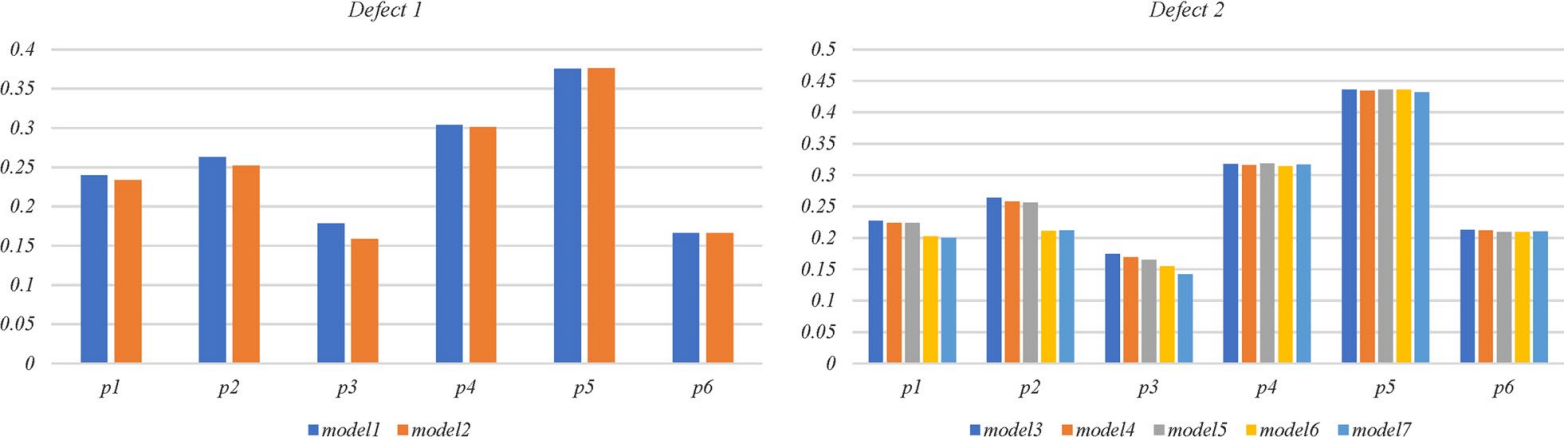
Stresses at 6 points on the midcourt line of the medial and lateral plateau (MPa). P: point

Stresses at the 6 points on the midcourt line of the medial and lateral plateaus are shown in Fig. 7. All stresses measured were within the normal range (0.1–2.8 MPa) [18]. The stresses of the medial plateau in model 7 (0.14–0.21 MPa) were significantly lower than those in model 3 (0.17–0.26 MPa, *p* = 0.038), model 4 (0.17–0.26 MPa, *p* = 0.04) or model 5 (0.16–0.26 MPa, *p* = 0.046). However, no other statistically significant differences were found (*p* > 0.05).

### Tibial tray

**Fig. 8:**
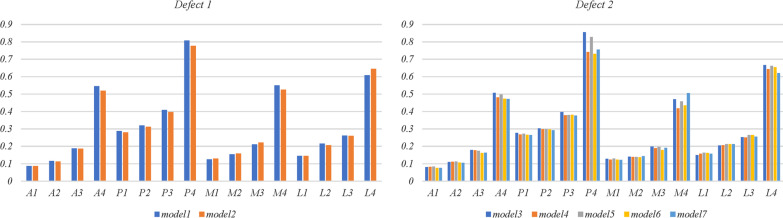
Stresses (MPa) at 16 points on the surface of cancellous bone in the medullary cavity. A: anterior; P: posterior; M: medial; L: lateral

Stresses at 16 points (anterior, posterior, medial, and lateral) on the surface of cancellous bone in the medullary cervicitis of all models are shown in Fig. 8. A stress point less than 0.1 MPa was found on the anterior surface of cancellous bone in all models, where local stress shielding may occur. The stresses on the surface of cancellous bone in the medullary cervicitis in model 6 (0.08–0.73 MPa) were significantly lower than those in model 5 (0.08–0.83 MPa, *p* = 0.037). All the other stresses measured were within the normal range (0.1–2.8 MPa), and no statistically significant differences were found (*p* > 0.05).

### Stress focus spots around the screws

Stresses at the focus points that exist on the cancellous bone around the screws are shown in Table 3. All stresses were within the normal range (0.1–2.8 MPa) except in model 4 (2.91 MPa). In models with a 9% defect area and an 8 mm depth, the use of one vertical screw resulted in significantly lower focused stress (0.45 MPa) than one oblique screw (1.72 MPa). In models with an 18% defect area and a 15 mm depth, higher stresses were found with the use of one screw (2.42 MPa and 2.91 MPa, respectively) than that two (2.22 MPa, 1.63 MPa, and 1.37 MPa, respectively).

**Table 3:** Stress focus spots around the screws (MPa)

	Screw 1	Screw 2
Model 1	1.72	
Model 2	0.45	
Model 3	2.42	
Model 4	2.91	
Model 5	1.92	2.22
Model 6	1.63	1.11
Model 7	0.42	1.37

## Discussion

Based on the FEA results, we found that (1) in defect 1, stresses on the surface of the cancellous bone were significantly lower when the screw was inserted vertically; (2) in defect 2, compared to one screw or two oblique screws, stresses on the surface of cancellous bone in defect and medial tibia surface were significantly lower when at least one vertical screw was inserted. We also compared the stress focus spots around the screws and found that (1) stress is significantly lower when one vertical screw was used to fill in small defects (defect area < 12%, depth < 10 mm); (2) higher stress occurs when only one screw or two oblique screws are used to fill a large defect (defect area > 12%, depth > 10 mm), which may destroy the cancellous bone around the screws; (3) either two vertical or two screws where one is vertical, and one oblique significantly reduces the stresses around them.

There are many types of basic reconstruction methods to treat defects of the tibial plateau in TKA [19]. For defects with a depth less than 10 mm, bone defects can be completely removed by adding bone resection and downsizing and lateralizing the tibial component without requiring further procedures [20]. However, excessive osteotomy may damage ligamentous structures, increase the stress on the proximal tibia, and require a thicker tibial insert [21]. Traditionally, the cement-only technique was used to fill defects less than 5 mm in depth after proximal tibial resection [19]. In all defects more than 5 mm, the cement-screw technique was suggested [22]. Compared to the cement-only technique, added screws greatly enhance the strength of the cement and reduce the possibility of the prothesis loosening [6]. For defects more than 5 mm, metal augmentation is another choice, which can have immediate stability and effective support for stable biomechanics [11]. However, metal augmentation is greatly limited due to the high price, limited shape, and thickness. In contrast, the cement-screw technique simplifies the operation, shortens operating time, and saves costs [7]. These advantages further promote the development of the cement-screw technique. The cement-screw technique is used to repair uncontained bone defects of the tibia in our clinical practice because of its many advantages.

Since being introduced into the clinic, many studies have demonstrated the efficacy of the cement-screw technique. In 1986, Ritter et al. [1] published a report on 57 cases utilizing screws and cement to fill tibial defects during TKA and obtained satisfactory short-term results. With an additional follow-up period of up to 13 years, they found that there were no signs of failure [6]. Berend et al. [4] performed 14,686 primary TKAs between December 1988 and February 2010; 256 patients received screws and cement for tibial defects. Compared to knees without screws, knees receiving tibial screws had significantly worse tibial bone quality preoperatively but equivalent survival probabilities at the last follow-up. From March 2018 to March 2019, Ozcan et al. [10] performed the cement-screw technique on 37 knees of 28 patients with high body mass index (BMI) (> 30 kg/m^2^). They also obtained satisfactory mid-term clinical outcomes and showed the efficiency of the cement-screw technique, even in high BMI patients. Despite its great success, the cement-screw technique may not be simple as it involves a lot of debate, such as the optimal number and angle of screws. Previous studies suggested that the number of screws depended upon the magnitude of the defect and its depth [1, 4]. However, for the angle, they did not describe the details. Berend et al. [4] suggested that screws should be inserted parallel to the proximal tibial cortex. In contrast, Liu et al. [8] inserted the screws perpendicular to the upper surface of the tibial plateau. Recently, Zheng et al. [9] performed an FEA to assess the differences between screw number and angle in TKA and showed that the number should be moderated for different defects. For the angle of the screws, they thought that vertical ones could achieve better stability than oblique ones. However, whether the screw was parallel to the proximal tibial cortex or not has not been described. Further, only one oblique screw was used in one model, and the analysis with two was absent.

The current incidence of periprosthetic fracture worldwide following primary TKA is believed to be in the range of 0.6–3.0% [23]. Because of alterations in the material properties of the bone as a result of aging, and thinning of the cortical bone structure, patients with osteoporosis may be particularly at risk of periprosthetic fracture following TKA [24]. Although stress fracture of the medial tibial plateau has been reported more after unicompartmental knee arthroplasty, it may occur after TKA as well [25, 26]. It's worth noting that most periprosthetic fracture was initiated at a cutting block pinhole site with varus collapse of the tibial component [26, 27]. Therefore, both reduced stresses and the angle of screws were very important to escape the stress fracture. The screw is needed not only to distribute the stresses, but also to transmit them effectively to the distal tibia.

There are some limitations associated with our study. First, this study is an FEA with models, and we did not consider screw interference with soft tissue that may occur in vivo. Second, we only compared two common angles reported in previous studies and did not consider the other angles, but it can be a good reference for clinical practice. Third, the cancellous screws used were 4 mm in diameter and 14 mm in length that is used in our clinical practice, and different diameters, materials, or lengths may have different effects on the stresses. Fourth, the models used in this study were based on a healthy patient with no deformity and good bone quality, which does not represent real occurrences in patients needing TKA. Finally, in clinical practice, the interactions of bone and screw include press-fit lock and friction, we only considered the frictional interaction and set a frictional coefficient of 0.3 between bone and screw based on previous studies. The knee prothesis used was cemented and fixed-bearing and we set fully bonded for the other contact pairs. Although the finite element model cannot close to real bone-screw interaction completely, it can provide some basic reference for future clinical practices. We believe this study may provide surgical guidance for performing TKA on patients with tibial bone defects.

## Conclusions

Screws perpendicular to the upper surface of the tibial plateau can achieve better stability than screws parallel to the proximal cortical bone of the tibia, either for one or two screws. If two screws cannot be inserted vertically, one vertical and one oblique are acceptable.

## Data Availability

The datasets used and/or analyzed during the current study are available from the corresponding author on reasonable request.

## Abbreviations

TKA: Total knee arthroplasty
FEA: Finite element analysis
CT: Computed tomography
3D: Three dimensional
BMI: Body Mass Index
